# Relationship between early-career collaboration among researchers and future funding success in Japanese academia

**DOI:** 10.1371/journal.pone.0277621

**Published:** 2022-11-11

**Authors:** Sho Tsugawa, Takuya Kanetsuki, Junichi Sugihara

**Affiliations:** 1 Faculty of Engineering, Information and Systems, University of Tsukuba, Tsukuba, Ibaraki, Japan; 2 BIOIMPACT Co., Ltd., Shinjuku, Tokyo, Japan; Universitat de Barcelona, SPAIN

## Abstract

Academia is becoming more and more competitive, especially for young scientists, so it is important to understand the factors that affect success in academic careers. To survive in academia, it is crucial to obtain research funding. Previous studies have investigated factors that affect the funding success of researchers. In this paper, we focus on research collaboration structure as a factor affecting funding success. More specifically, we investigate the effects of participation in joint research projects, number of joint research projects, and centrality in the collaborative network on the future funding success of junior researchers in Japan. Our results show that participation in joint research projects and the number of such projects significantly affect the future funding success of junior researchers. Furthermore, the median number of years of funding received by researchers involved in joint research projects was found to be about 1.5 times greater than that of researchers not involved in joint research projects, and the average amount of research funding received after 10 years is about 2–4 times more, suggesting that researchers with collaboration ties with other researchers in the early stages of their career tend to be more successful in the future.

## Introduction

Much attention has been devoted to the science of science, which is a field that aims to advance science and technology by exploring questions such as what kinds of research environments have led to scientifically important discoveries and what kinds of researchers or research groups have achieved success [1, 2]. There are several research topics in the science of science, which include analyses of the structure of co-authorship networks and citation networks as well as their changes over time [3–5], analysis of the factors of researchers’ success [6], methods to predict which researchers will be successful in the future [7, 8], analysis of the characteristics of research groups that publish high-impact research results [9], and modeling citation dynamics [10]. Such research is expected to contribute to the presentation of various career paths to researchers, more effective evaluation of research, better design of science and technology policies, and the discovery of scientifically important fields [1].

Because obtaining research funding is important for academic researchers to continue their research activities, many studies have investigated the factors that affect researchers’ funding success [11–14]. As competition among academic researchers has intensified around the world, continually obtaining external funding from the government, corporations, foundations, and other sources has become a critical factor in the ability to continue academic research activities. This situation has led to increased interest in analyzing what types of researchers are successful at obtaining funding. Previous studies have found that gender [11, 15, 16], the interdisciplinarity of the research field [12], and success in obtaining funding early in one’s career [13, 14] affect the ability to obtain research funding in the future.

In this study, we analyze the relationship between collaborative research early in one’s research career and the ability to obtain funding in the future. In today’s increasingly complex fields of natural science and engineering, it is rare for an individual researcher to conduct research alone. Indeed, the team science conducted collaboratively by multiple researchers is suggested to be a key for significant scientific findings [17]. For instance, it is shown that Nobel prize winners have spent their early years working in larger teams than others [18]. In contract, previous studies suggest *the rich get richer* phenomena in the scientific collaboration network [19]. Namely, researchers who have many collaborators tend to get more collaborators in the future. These findings suggest that for researchers, early-career collaboration with other researchers is a key for their success. In this paper, we particularly focus on the early-career collaborations of researchers in joint research projects in their future funding success. Previous studies have examined the effect of co-authorship on individual researchers’ future success in publishing papers [8, 20–26]. However, to our knowledge, no studies have examined how researchers’ future funding is affected by their participation in projects in which funding was jointly obtained by the collaborating researchers. In addition, previous studies examining factors of funding success [11–14] have not examined joint research as a factor for success.

The specific research questions addressed by this paper are as follows.

- **(RQ1)**: What is the relationship between participating in a joint research project early in one’s career and the ability to obtain research funding in the future?
- **(RQ2)**: What is the relationship between the number of joint research projects a researcher participates in early in their career and their ability to obtain research funding in the future?
- **(RQ3)**: What is the relationship between a researcher’s centrality [27] in collaborative networks early in their career and their ability to obtain research funding in the future?

To answer these questions, we analyze data on competitive research funding by public entities in Japan over a period of 18 years. Using a research funding database, we extract data on young researchers who obtained research funding for the first time in a given year. We then analyze the relationship between the collaboration patterns of those young researchers and their ability to obtain funding in the future.

## Methodology

### Data

We utilized a dataset on research grants from the Japanese government. The data in the dataset were collected by BioImpact Co., Ltd., to which two of the authors belong, and made available for this study. The dataset covers major funding programs in Japan, including grants from the Japan Society for the Promotion of Science (JSPS), Japan Science and Technology Agency, Japan Agency for Medical Research and Development, Ministry of Health, Labour and Welfare, New Energy and Industrial Technology Development Organization, and Ministry of Education, Culture, Sports, Science and Technology. The dataset contains information about the principal investigator (PI) and co-investigators (Co-Is) of each project, the scientific domain of the project, and the amount of grant money for each fiscal year (FY). The original data used in this study are available on the website research-er.jp (https://research-er.jp/ in Japanese). The dataset contains 560,308 accepted research projects during the period FY2000–FY2017. Each project has a PI and may also have had Co-Is who are also involved in the project. For each project, the amount of grant money for each FY is available. Researchers (i.e., PI and Co-Is) in our dataset belong to one of the following three scientific domains: “humanities and social sciences,” “science and technology,” and “medicine and life science.”

For this study, we extracted junior PIs who had just started their academic career. The most common funding for researchers in Japanese academia is JSPS KAKENHI (https://www.jsps.go.jp/english/e-grants/index.html). JSPS KAKENHI provides several types of funding programs that only young researchers can apply for. Researchers under the age of 40 can apply for Grant-in-Aid for Young Scientists (A) and (B), whereas researchers who have just started their professional research career can apply for Grant-in-Aid for Research Activity Start-up. Thus, researchers who receive these programs for the first time were extracted as the target PIs for the analyses. We investigated how the target PIs who received their first funding obtained subsequent funding over time. To examine the long-term funding success of researchers, we need to observe them for a certain period of time (e.g., 10 or more years) after their first funding. Therefore, in the following analyses, we used mainly the data on young PIs extracted from FY 2004. We also used the data on young PIs extracted from FY 2005 and 2006 (Supporting information) to validate the findings from the main data. PIs extracted from after FY 2007 were not used because observation periods after their first funding would be too short. Note that data during FY 2000–2003 were used only to ascertain whether the target PIs had never obtained research funding in the past. The detailed procedures to extract target PIs for FY 2004 were as follows. We first extracted PIs from above KAKENHI programs accepted in FY 2004. From these PIs, those who had obtained any research funding during FY 2000–2003 were removed. Then, remaining PIs were target PIs for the following analyses. The number of extracted PIs were 3,061, 3,511, and 4,367 for FY 2004, 2005, and 2006, respectively. Our dataset is available at https://doi.org/10.6084/m9.figshare.20647608.

### Measures

As measures for funding success of researchers, we used the number of funded years and the amount of grant money. It is difficult to determine what constitutes funding success for PIs because it depends heavily on several factors, including their field and research theme. We consider that it is important for PIs to continuously obtain research funding to operate their laboratories. Therefore, we used the number of years in which they received research grants as a PI in order to measure the continuity of research funding. The number of funded years for a researcher is defined as the number of years where the researcher has at least one project as a PI. We use this measure to evaluate how the length of the period with no grant is small. The amount of grant money required differs according to the scientific domain, but in general, the size of the research grant can be another measure of funding success. For each year, we calculated the total amount of grant money the target PIs obtained. Also, we determined the total number of years in which the PIs received any research grant as a PI. Note that we did not include research projects in which the target PIs were a Co-I.

As factors that can affect funding success, we focused on the collaboration patterns of the PIs. We first examined the effects of the existence of collaborators on the future funding success of a PI (**RQ1**). The target PIs had at least one research project of their own (i.e., a project funded by a JSPS KAKENHI Grant-in-Aid for Young Scientists). JSPS KAKENHI Grant-in-Aid for Young Scientists is a funding program for a single PI (i.e., there are no Co-Is in the program). In contrast, the young PIs could be a Co-I of other researchers’ projects or they could have Co-Is in their other projects. We investigated the difference in funding success between PIs who had any collaborations in the first three years of their career and those who did not. A PI is considered to have had a collaboration if they were a Co-I of a project run by another PI or if there was a Co-I on their own project(s).

For the target PIs who had collaborations in their first three years, we also investigated their collaboration patterns. To quantify the collaboration patterns, we used the number of projects (**RQ2**), degree centrality [27], and betweenness centrality [27] (**RQ3**). The number of projects for a researcher is defined as the number of projects that they were involved with either as PI or Co-I. For young PIs of FY *y*, the data during FY *y* and *y* + 2 were used to obtain the number of projects. It is expected that researchers who have more collaborations tend to be more successful. The degree centrality and betweenness centrality of a PI are measures of their influence in the collaborative network. Following [28], we constructed a collaborative network *G* = (*V*, *E*) of researchers. Node *v* ∈ *V* represents a researcher, and link (*u*, *v*)∈*E* represents researchers *u* and *v* who belong to at least one project in common. To calculate these measures, we used data for three years. That is, for PIs who started their career in FY *y*, we used data from FY *y* to FY *y* + 2. Using the constructed collaborative network *G*, we calculated the degree and betweenness centralities of the nodes. The degree centrality of a node is defined as the number of links with the node. Thus, PIs who have many collaborators tend to have high degree centrality. The betweenness centrality of a node is defined as the number of shortest paths passing through the node. Thus, PIs who bridge researchers in different fields tend to have high betweenness.

## Results

In this section, we examine the effects of early-career collaborations on the future funding success of young PIs. In what follows, we show only the results for new PIs in FY 2004; the results for FY 2005 and FY 2006 are shown in the S1–S8 Figs. We confirmed that similar results were obtained across different years.

**Fig 1:**
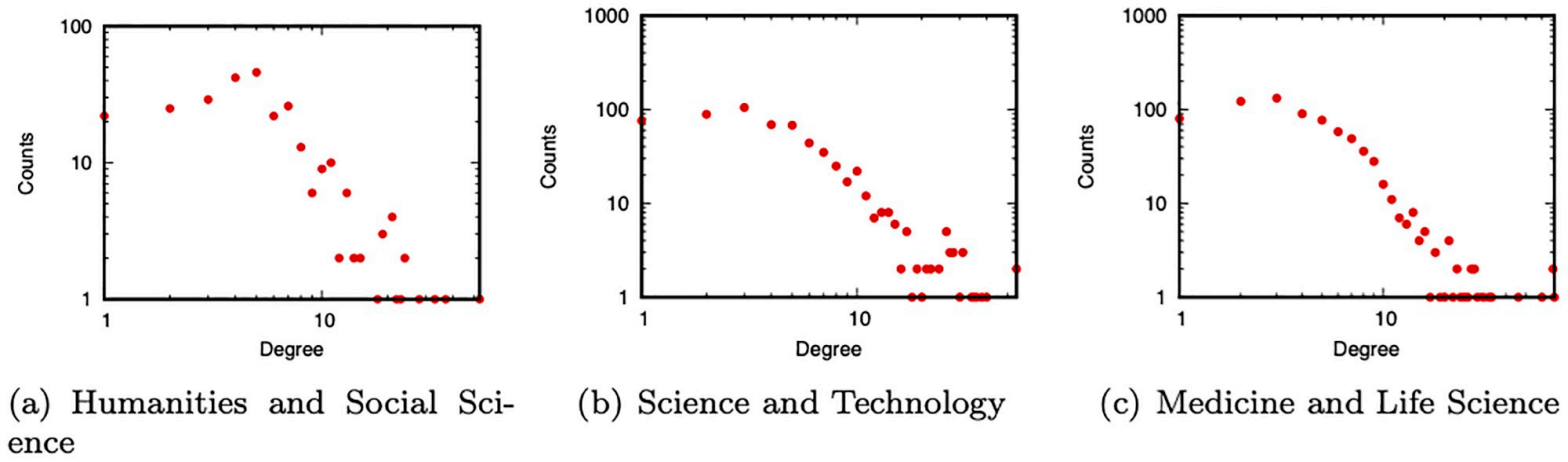
Distributions of degree centrality (FY 2004).

**Fig 2:**
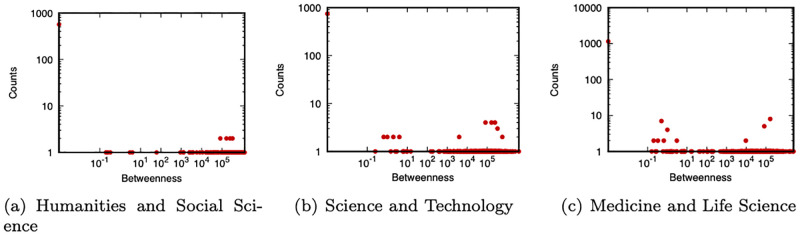
Distributions of betweenness centrality (FY 2004).

**Fig 3:**
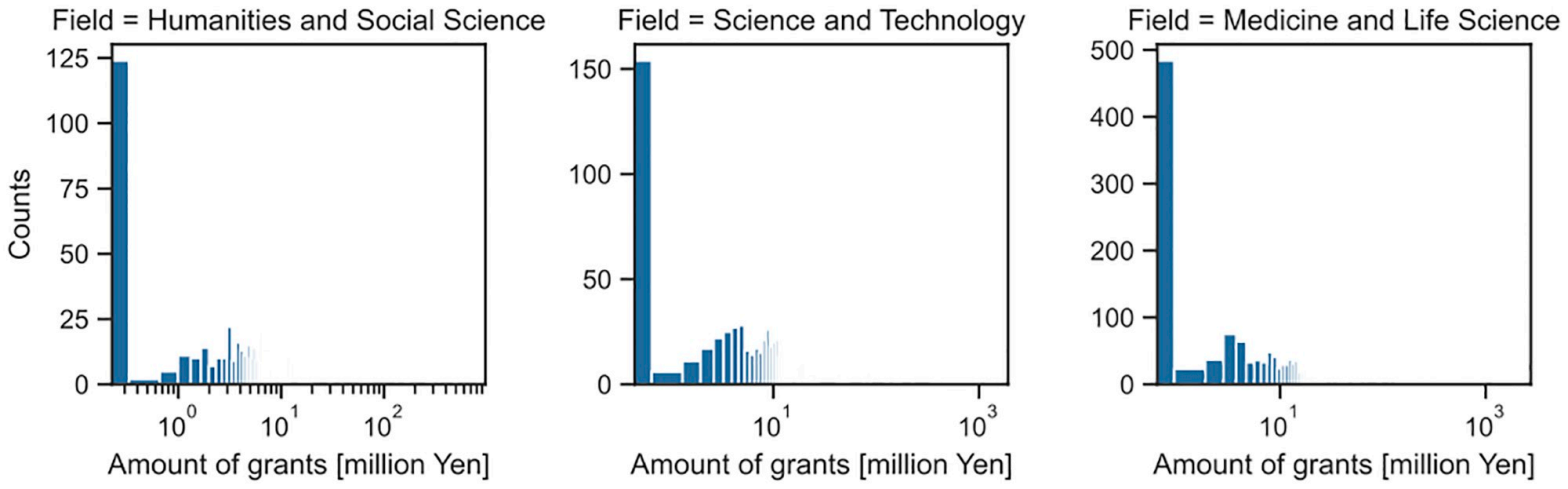
Distributions of the total amount of obtained grant of target PIs during FY 2004–2017.

Before examining the results, we show some basic statistics about our dataset for FY 2004. Basic statistics of the collaboration network *G* are shown in Table 1. Distributions of degree centrality and betweenness centrality of the target PIs are shown in Figs 1 and 2, respectively. The number of samples (i.e., the number of the target PIs) for each category is shown in Table 2. Distributions of the total amount of grant money obtained by target PIs during FY 2004–2017 are shown in Fig 3.

**Table 1:** Statistics of the collaboration network (FY 2004).

Num. of nodes	96,173
Num. of links	436,572
Average degree	9.08
Clustering coefficient [29]	0.72
Average shortest path length	6.64

**Table 2:** Number of target PIs in the dataset (FY 2004).

	w/ collaborators	w/o collaborators
humanities and social sciences	254	404
science and technology	603	301
medicine and life science	719	700

### Effects of the existence of collaborations (RQ1)

**Fig 4:**
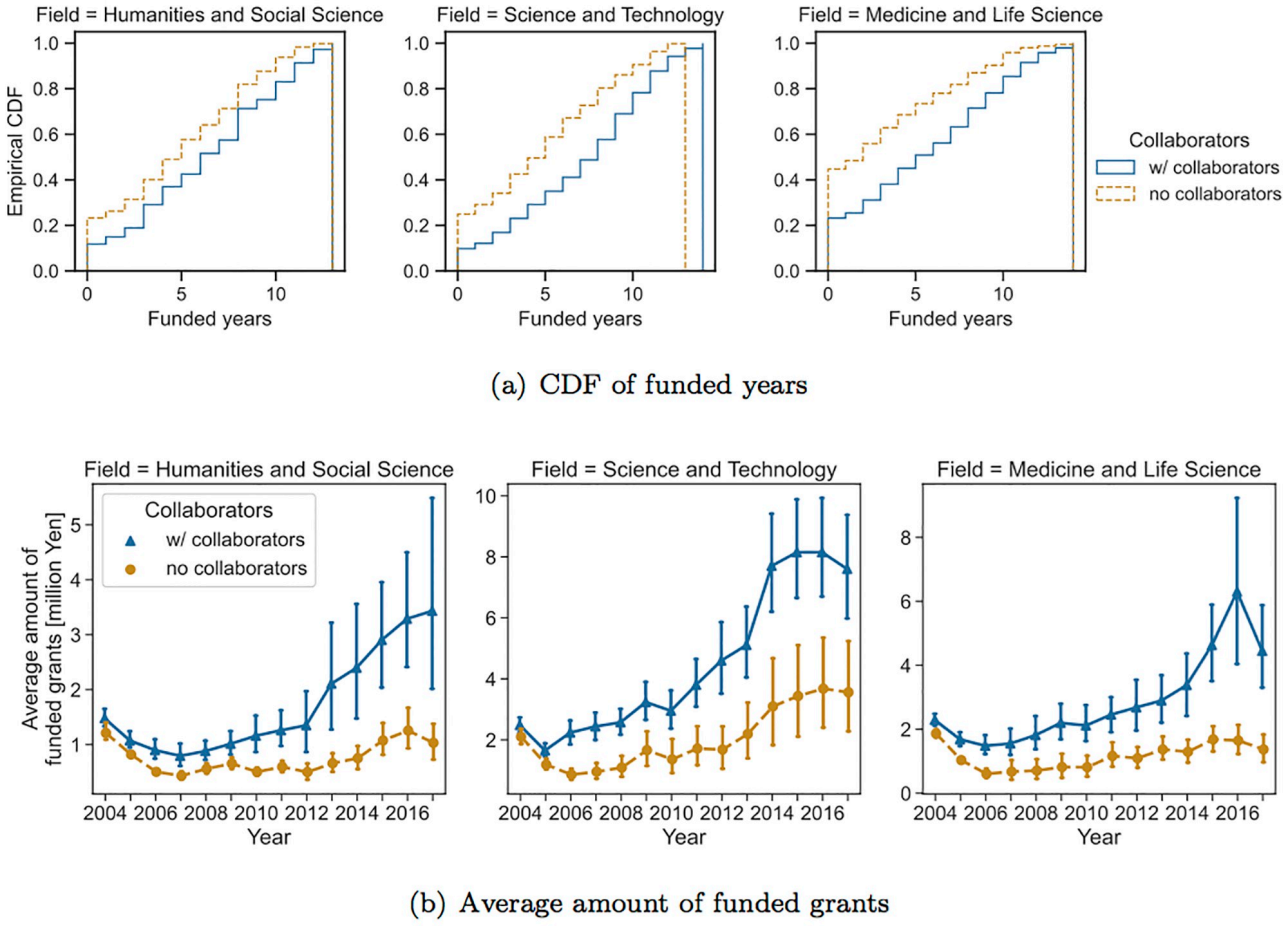
Comparison of measures of funding success between PIs with collaborators and those without (FY 2004).

We first compare researchers who had no collaborators with those who had at least one collaborator. For FY 2004 data, 1,576 PIs had collaborators, and 1,485 did not. Fig 4 shows the empirical cumulative distribution functions (CDFs) of the number of funded years for each scientific domain as well as the average amount of funded grants for each year. The error bars show the 95% confidence intervals. Although the target PIs had at least one project (i.e., project funded by JSPS KAKENHI Grant-in-Aid for Young Scientists), the number of funded years does not include the years for the projects.

This figure shows that for all three scientific domains, PIs who had at least one collaborator were able to obtain research funding for a greater number of years compared with PIs who did not have any collaborators. For instance, in the field of medicine and life science, approximately 40% of the target PIs who did not have any collaborators did not receive any research funding during the observation period, whereas for PIs with collaborators, the percentage of researchers who did not receive any research funding was approximately 20%. The Mann–-Whitney U test showed that there was a significant difference in the number of funded years between researchers with collaborators and those without across all domains (*p* < 0.01). In terms of the amount of grants, the results show that PIs who have collaborators receive more money than those who do not. In particular, as time passes, the gap between researchers who have joint research projects and those who do not widens, reaching a difference of about 2–4 times after about 10 years. Note that large error bars, particularly for PIs with collaborators, shown in the figures of the average amount of grant money are due to the existence of PIs who obtain extremely larger amount of grant than other researchers (see Fig 3).

### Effects of the number of research projects (RQ2)

**Fig 5:**
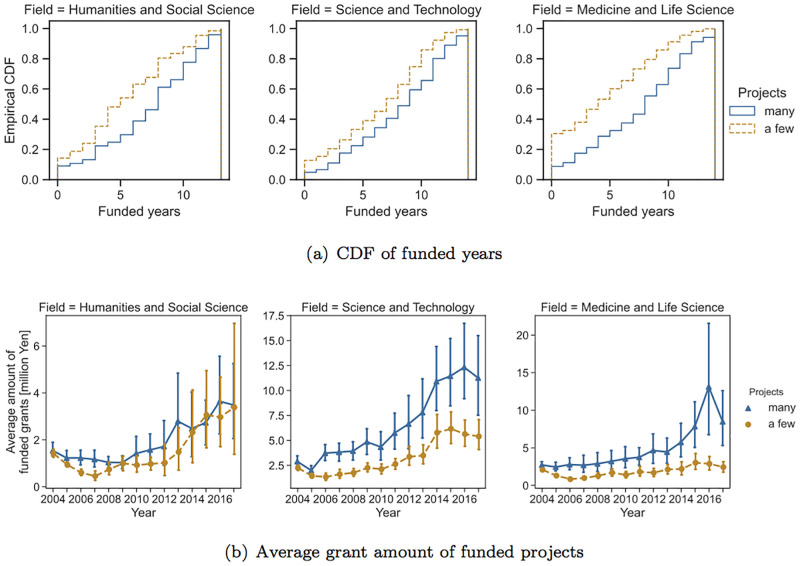
Comparison of measures of funding success between PIs with many projects and those with a few projects (FY 2004).

We next look at the effects of the number of research projects that PIs are involved with in their first three years on their future funding success. In the following analyses, we used only the data for PIs who had at least one collaborator. Fig 5 compares the empirical CDFs of the number of funded years and the average grant amount for each year between PIs with many projects and those with a few projects. PIs whose number of projects was greater than the median number of projects among PIs in the same scientific domain were classified as PIs with many projects, while the rest were classified as PIs with a few projects. The median number of projects was 2, 3, and 3 for the humanities and social science, science and technology, and medicine and life science fields, respectively.

Fig 5 shows that young PIs with many projects tend to obtain grants for a longer period of time and receive larger grant amounts compared PIs with a few projects. The Mann–-Whitney U test showed that there was a significant difference in the number of funded years between PIs with many projects and those with a few projects across all scientific domains (*p* < 0.01). For instance, in the field of medicine and life science, approximately 30% of the target PIs with a small number of projects did not receive any research funding during the observation period, whereas the percentage of PIs with many projects who did not receive research funding was less than 10%. In the field of humanities and social science, the difference in the grant amount of funded projects between PIs with many projects and those with a few projects was not clear, but there was a difference in the number of funded years.

To further investigate the difference between PI with many projects and PI with a few projects, we counted the number of PI projects and Co-I projects for each PI. A PI project for a researcher is a project in which he/she involved as PI, and a Co-I project for a researcher is a project in which he/she involved as Co-I. Note that the number of projects for a researcher is the sum of the number of PI projects and the number of Co-I projects. Table 3 compares the number of PI projects and the number of Co-I projects between researchers with many projects and researchers with a few projects. Table 3 shows that while the difference in the number of Co-I projects between *many* class and *a few* class is large, the number of PI projects is not so different between these two classes. Therefore, our results suggest that researchers who actively participate in many projects as Co-I will tend to get larger amount of grant money as PI in the future, which suggests the importance of early-career collaborations of researchers for their funding success.

**Table 3:** Mean number of PI projects and Co-I projects (FY 2004).

	PI projects		Co-I projects	
	a few	many	a few	many
Humanities and Social Science	1.02	1.45	0.98	2.35
Science and Technology	1.23	1.80	1.28	3.14
Medicine and Life Science	1.24	1.97	1.26	3.43

### Effects of centrality (RQ3)

**Fig 6:**
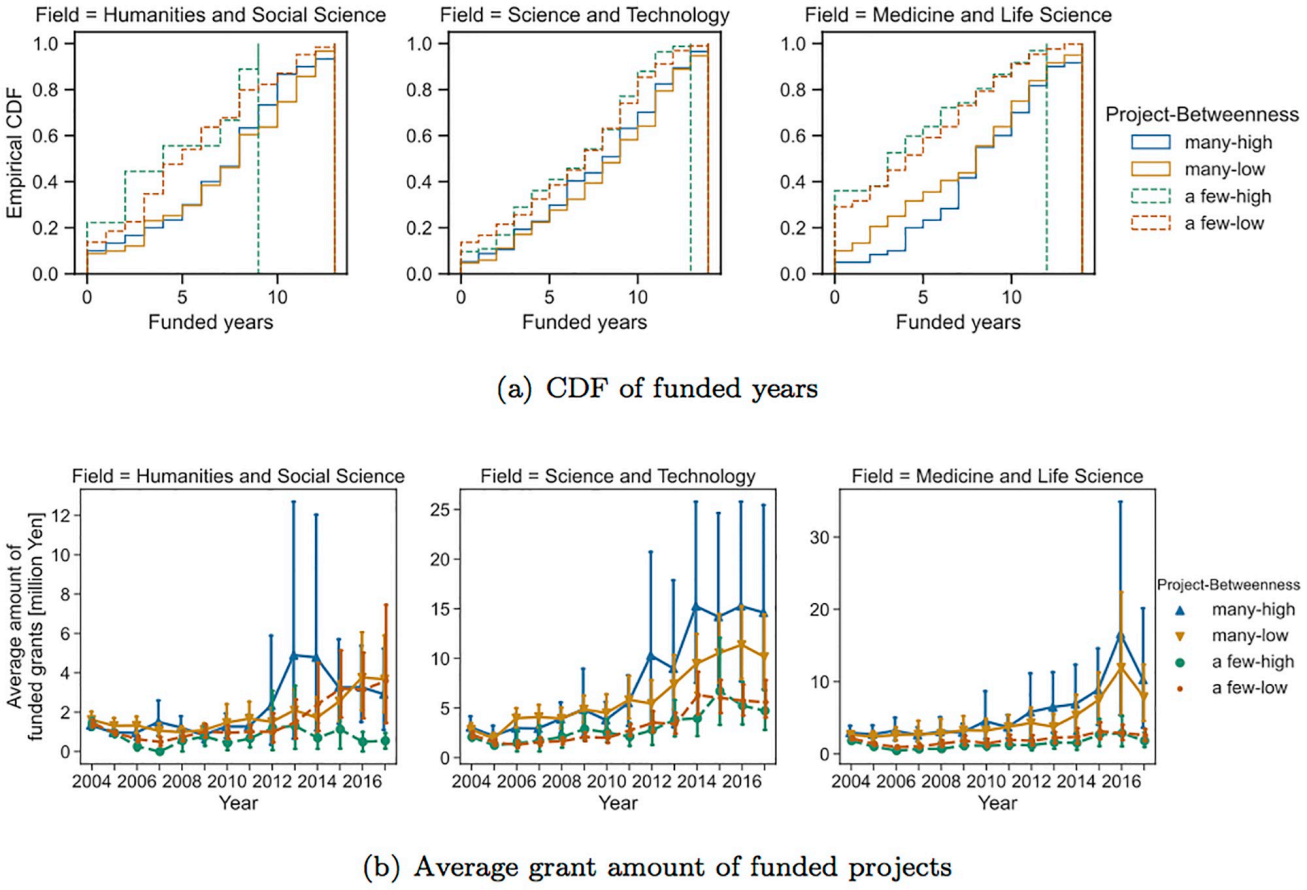
Comparison of measures of funding success between PIs with high-betweenness and those with low-betweenness (FY 2004).

Finally, we tackle the effects of centrality on the funding success of PIs. Specifically, we compare researchers with relatively high centrality and others. By definition, the number of projects a PI is involved with is highly correlated with their centrality. In other words, PIs with many projects tend to have high centrality. Taking into account this effect, we compare four classes of researchers: many projects and high centrality (many-high), many projects and low centrality (many-low), a few projects and high centrality (a few-high), and a few projects and low centrality (a few-low). Here, PIs with high centrality were those whose centrality was larger than the 3rd quartile of centrality. Fig 6 compares measures of funding success among these four groups of PIs based on the number of research projects and betweenness centrality.

**Fig 7:**
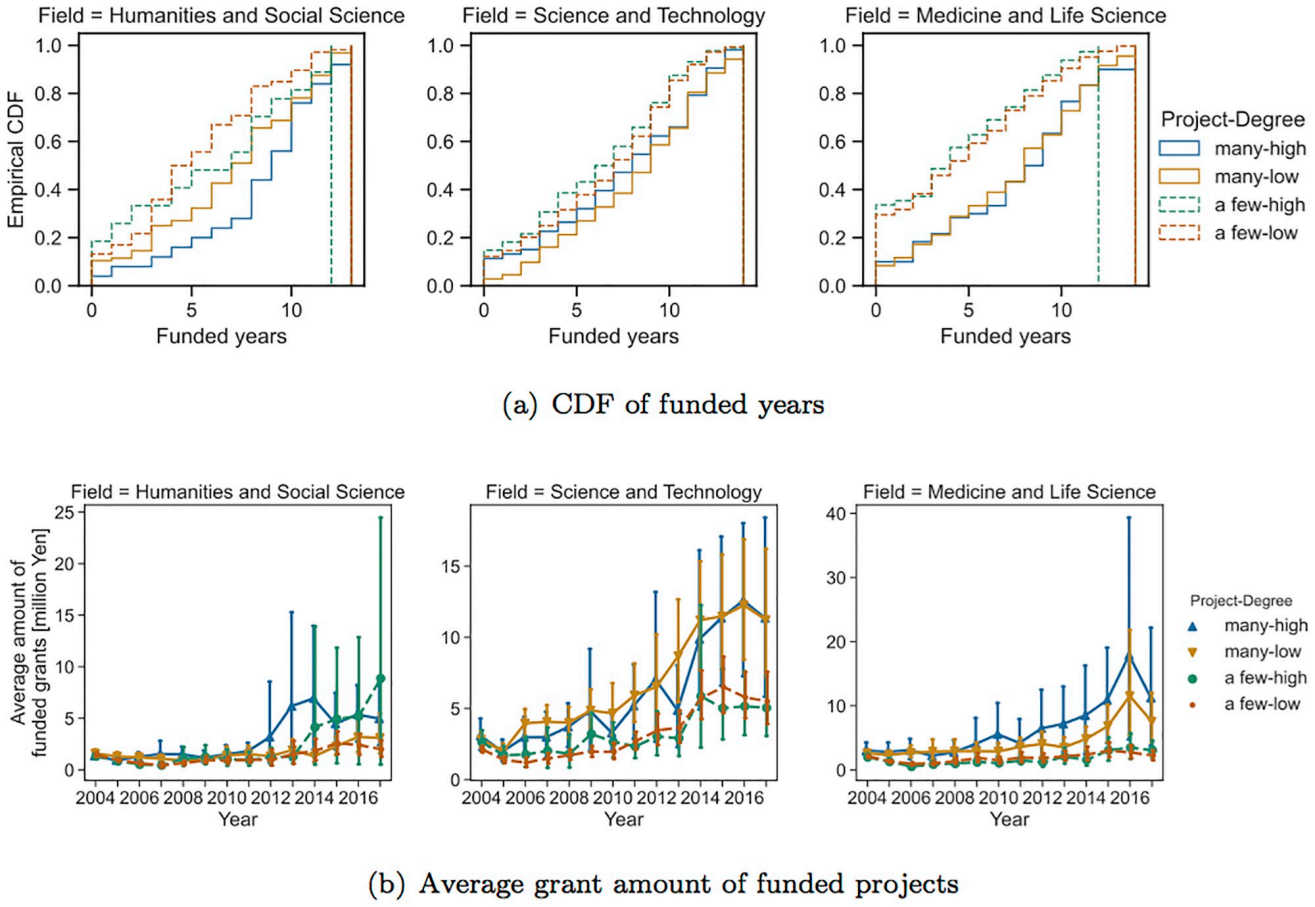
Comparison of measures of funding success between high-degree and low-degree PIs (FY 2004).

These results show that there was not a clear difference between the high-centrality and low-centrality groups. For medicine and life science, there was a difference in the grant amounts between the many-high and many-low groups. However, the effects of betweenness does not seem to be large. Fig 7 shows the same results for degree centrality, which showed similar tendencies with the results for betweenness centrality.

## Discussion

### Implications

The results of this paper’s analysis show that the amount of future research funding and the number of funded years significantly vary with early-career participation in a joint research project and the number of such joint projects. Previous studies have shown that obtaining research funding early in one’s career has an effect on obtaining research funding in the future [13, 14]. However, even among researchers with early-career success in obtaining research funding, the degree of subsequent success in obtaining funding significantly varies with early-career participation in a joint research project and the number of such joint projects. Approximately 30% of the JSPS KAKENHI grant applications considered in our analysis were approved, and the young PIs considered in our analysis were those whose applications were approved. The finding that, even for researchers who have passed such a competitive approval process, subsequent success in obtaining funding significantly varies according to participation in other joint research projects suggests that participating in joint research projects is important for obtaining funding in academia. As research topics in recent years have become increasingly complex, it has become more common for multiple researchers to collaborate on such research. We therefore consider that researchers actively engaging in joint research are more likely to receive research funding. It should be noted that there can be other factors affecting funding success than early-career collaboration. One interpretation of our results is that researchers capable of obtaining a large amount of funding tend to receive invitations to participate in joint research efforts. Another interpretation is that young researchers from influential laboratories engage in many joint projects with PIs from the same laboratories and are subsequently able to obtain continuous funding. To incorporate other factors into the analysis will be an important future work.

Another finding is that the amount of future research funding and the number of funded years do not significantly vary with centrality in the collaborative network. Although studies on a co-authorship network show that the centrality index is correlated with the degree of success in publishing papers [20, 30], this paper’s analysis finds only a weak effect. We consider that because centrality is strongly correlated with the number of joint research projects due to its definition, the variability of centrality is not large for researchers with a similar number of joint research projects, and as a result, differences between high-centrality and low-centrality groups are not substantially large. As seen in the average research funding for different years (Fig 6), in the medicine and life science field as well as the science and technology field, high-centrality groups tend to obtain slightly more research funding compared with low-centrality groups. Further investigation is needed to understand the effect of centrality. Moreover, using a network measure that is independent from the number of projects will be an interesting future task.

This paper’s results also imply that small difference between researchers in their early-career stages results in big difference between them in future funding success; that is, researchers who actively engaged in many research projects early in their career became increasingly successful, whereas those who did not found it more difficult to succeed. Previous studies have shown that researchers who were successful early in their career tended to be increasingly successful thereafter. Consequently, disparities among researchers became increasingly large [31]. From the standpoint of providing research opportunities to various researchers, it is considered important to have a scientific research policy that provides opportunities to researchers who were not able to participate in joint research projects early in their career.

### Limitations

We recognize that this paper’s analysis has some limitations. First, because the results are based on data from a Japanese population, it is not clear if they are generalizable to other countries with different research funding systems. Data from some countries have provided evidence supporting the existence of the Matthew effect in research funding [13, 14], but the generality of the effect of certain patterns of joint research on funding success needs to be investigated using different data. Second, this paper focuses on only one aspect of researchers’ success, namely, obtaining research funding; therefore, further analysis is needed to determine the effect of joint research on other aspects of success, such as the publication of papers. For instance, comparing the h-index as of 2004 between PIs with collaborators and those without will be useful for validating the effects of collaborations on their funding success. It is also interesting to compare future h-index between PIs with collaborators and those without to investigate the effects of collaborations on their publication success. Third, our analyses do not distinguish PIs who have already quit science and PIs who continue research but did not get government funding. Some of the PIs who did not get government funding might leave academia. In contrast, some other PIs might conduct research with funding sources other than government grants such as funds obtained for joint research with a private-sector company. Accordingly, in future work, we need to include researchers who conduct research with funding sources other than government grants, and investigate the characteristics of researchers who survive in academia and those who left academia. Forth, there can be other possible measures for quantifying funding success other than the measures used in this paper. In particular, since quantifying the funding continuity is not trivial, there could be suitable measures other than the CDF of the number of funded years. Lastly, this paper uses data for 2004–2006 to perform a long-term analysis, but there is a possibility that tendencies found in the data have changed since then. Further investigation is needed to determine what, if any, changes may have occurred over time.

## Conclusion

This paper analyzes the relationship between researchers’ early-career participation in joint research and their success in obtaining research funding in the future. More specifically, it considers participation by young researchers in Japan in joint research projects, the number of joint projects they participate in, and the centrality in the collaborative network, thereby analyzing the relationships between these factors and the amount of future research funding and the number of funded years. The results of the analysis show that researchers who participated in a joint research project tended to be more successful in obtaining future funding compared with those who did not; that the greater the number of joint projects the researchers participated in, the more successful they tend to be in obtaining future research funding; and that the centrality index for a collaborative network has a limited association with the ability to obtain research funding in the future. For researchers who participated in a joint research project early in their career, the median number of years with research funding and the average amount of funding obtained 10 years later were 1.5 times and 2–4 times larger, respectively, than those for researchers who did not participate in a joint research project early in their career. These results suggest the importance of early-career collaboration of researchers in their success.

## Supporting information

S1 Fig Comparison of measures of funding success between PIs with collaborators and those without (FY 2005). (TIF)

S2 Fig Comparison of measures of funding success between PIs with collaborators and those without (FY 2006). (TIF)

S3 Fig Comparison of measures of funding success between PIs with many projects and those with a few projects (FY 2005). (TIF)

S4 Fig Comparison of measures of funding success between PIs with many projects and those with a few projects (FY 2006). (TIF)

S5 Fig Comparison of measures of funding success between PIs with high-betweenness and those with low-betweenness (FY 2005). (TIF)

S6 Fig Comparison of measures of funding success between PIs with high-betweenness and those with low-betweenness (FY 2006). (TIF)

S7 Fig Comparison of measures of funding success between high-degree and low-degree PIs (FY 2005). (TIF)

S8 Fig Comparison of measures of funding success between high-degree and low-degree PIs (FY 2006). (TIF)
