# Supplementary figures and images for: Relationship between early-career collaboration among researchers and future funding success in Japanese academia
Source: PLoS One. 2022 Nov 11;17(11):e0277621. doi: 10.1371/journal.pone.0277621 (PMC9651575; doi:10.1371/journal.pone.0277621)

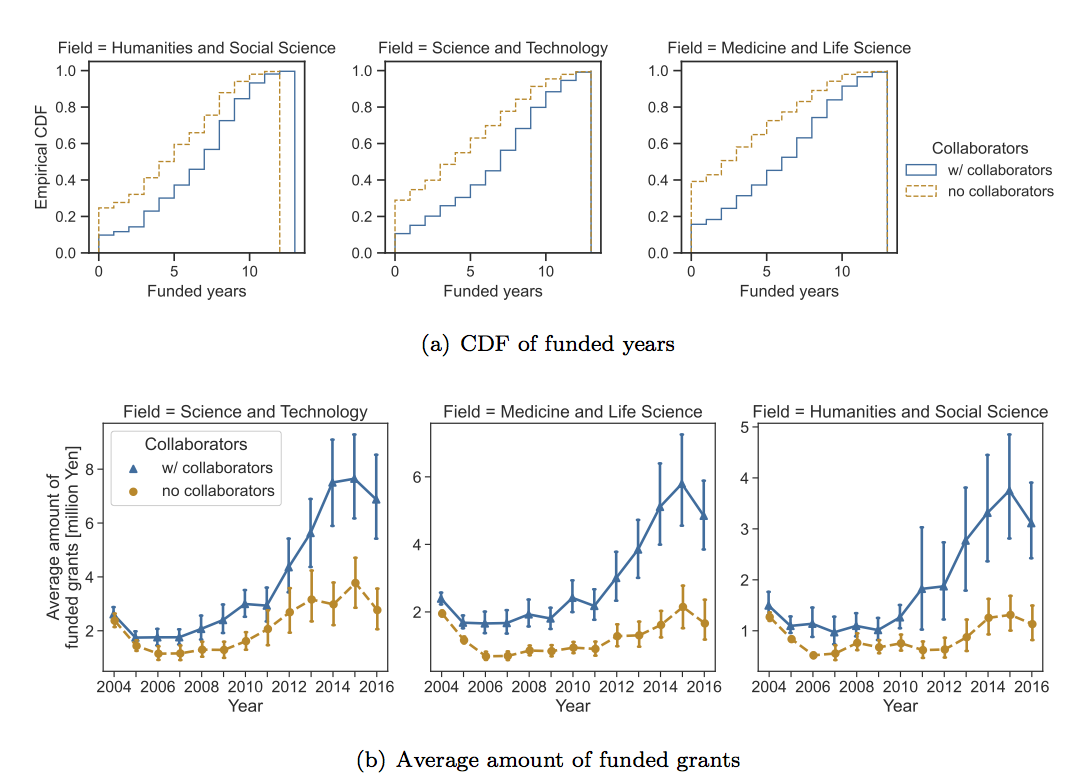

Supplement: S1 Fig — (TIF) [file pone.0277621.s001.tif]

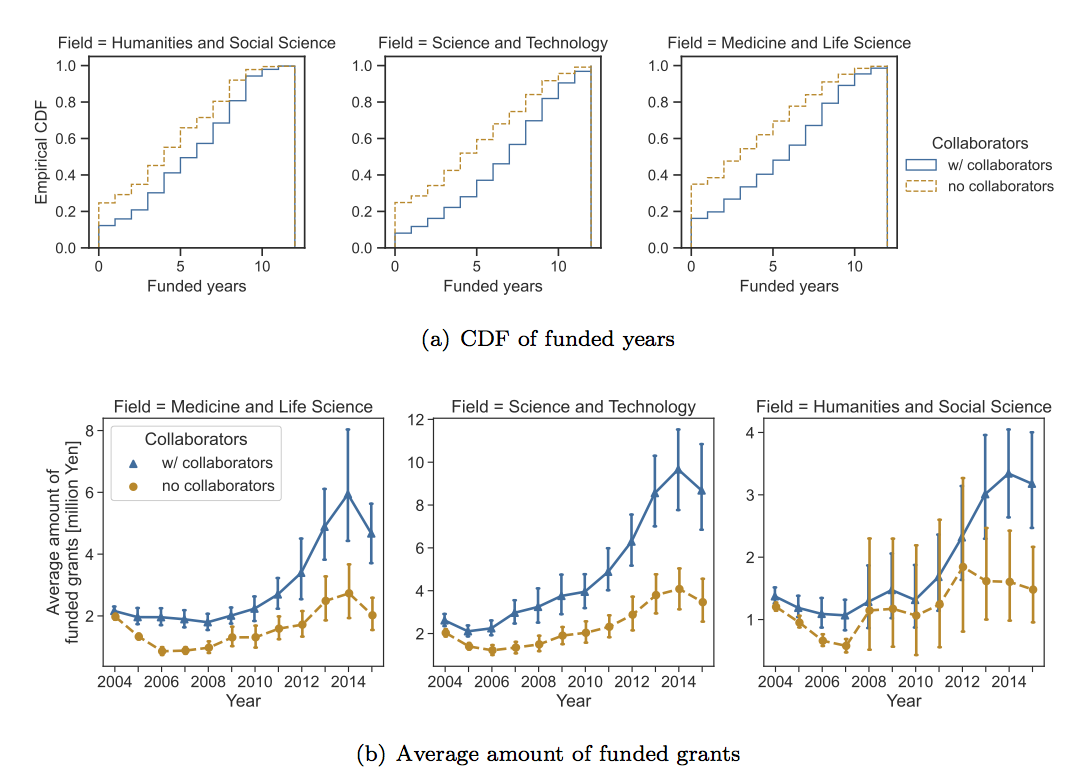

Supplement: S2 Fig — (TIF) [file pone.0277621.s002.tif]

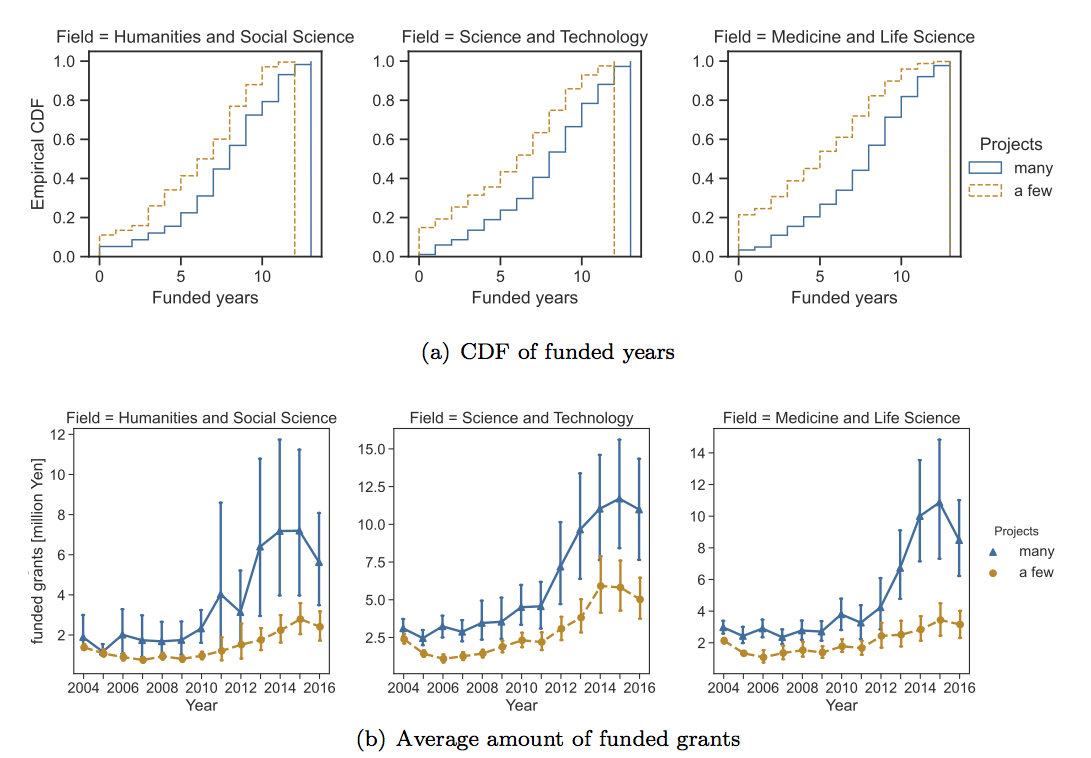

Supplement: S3 Fig — (TIF) [file pone.0277621.s003.tif]

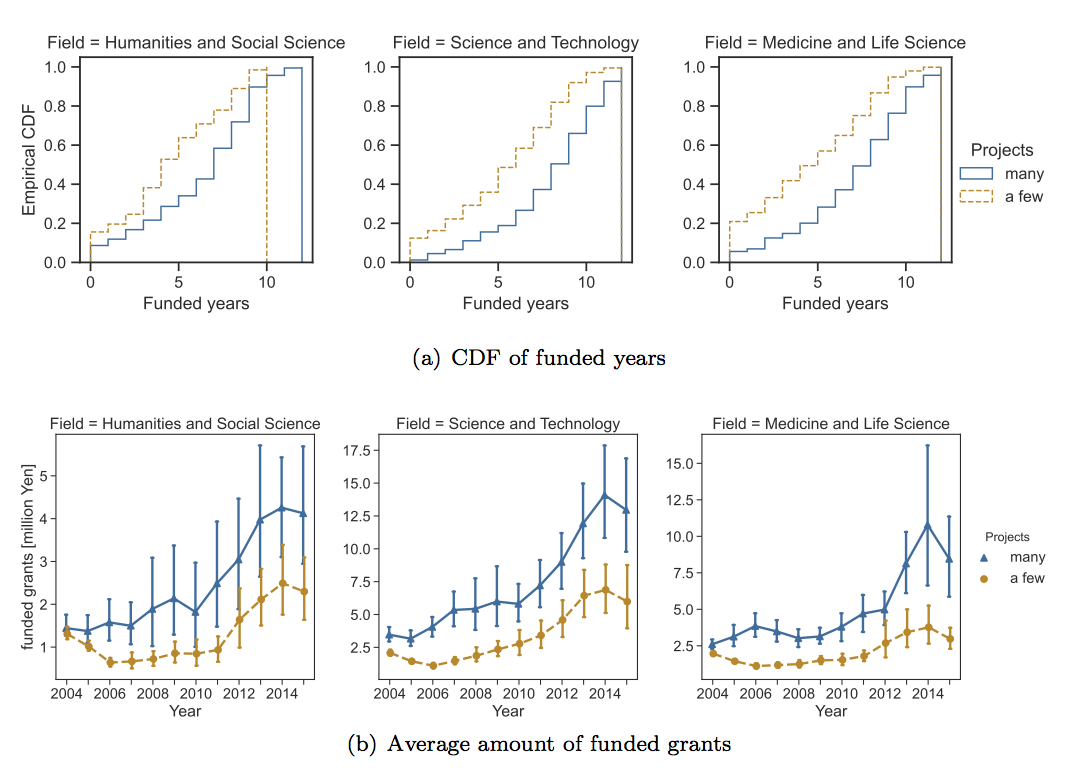

Supplement: S4 Fig — (TIF) [file pone.0277621.s004.tif]

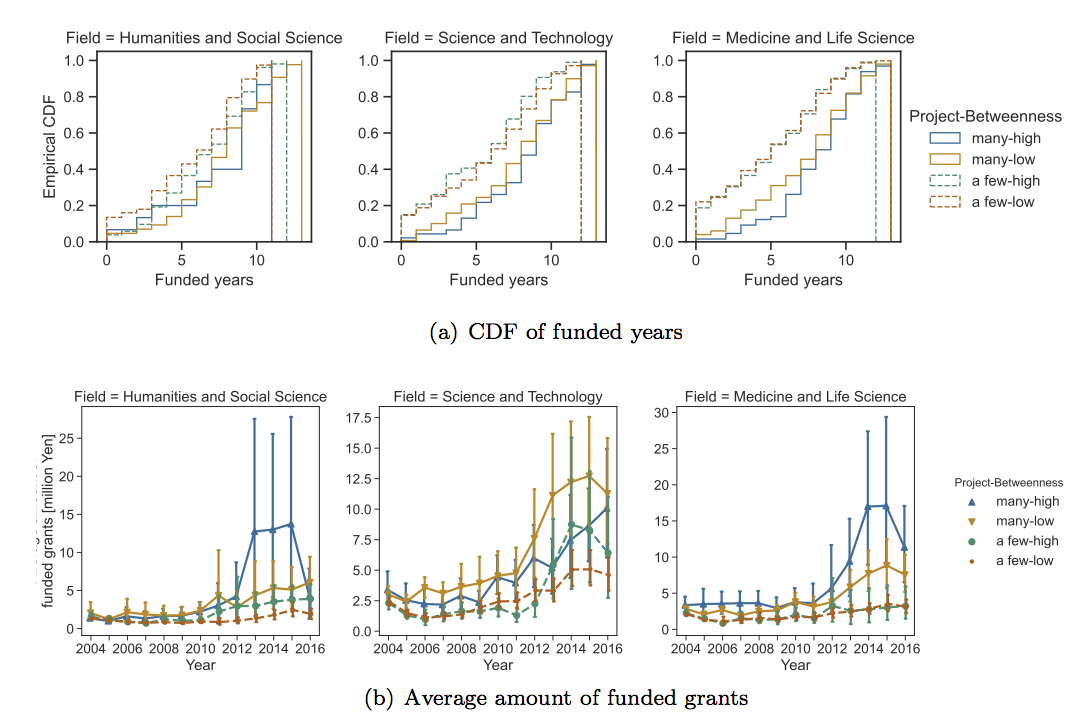

Supplement: S5 Fig — (TIF) [file pone.0277621.s005.tif]

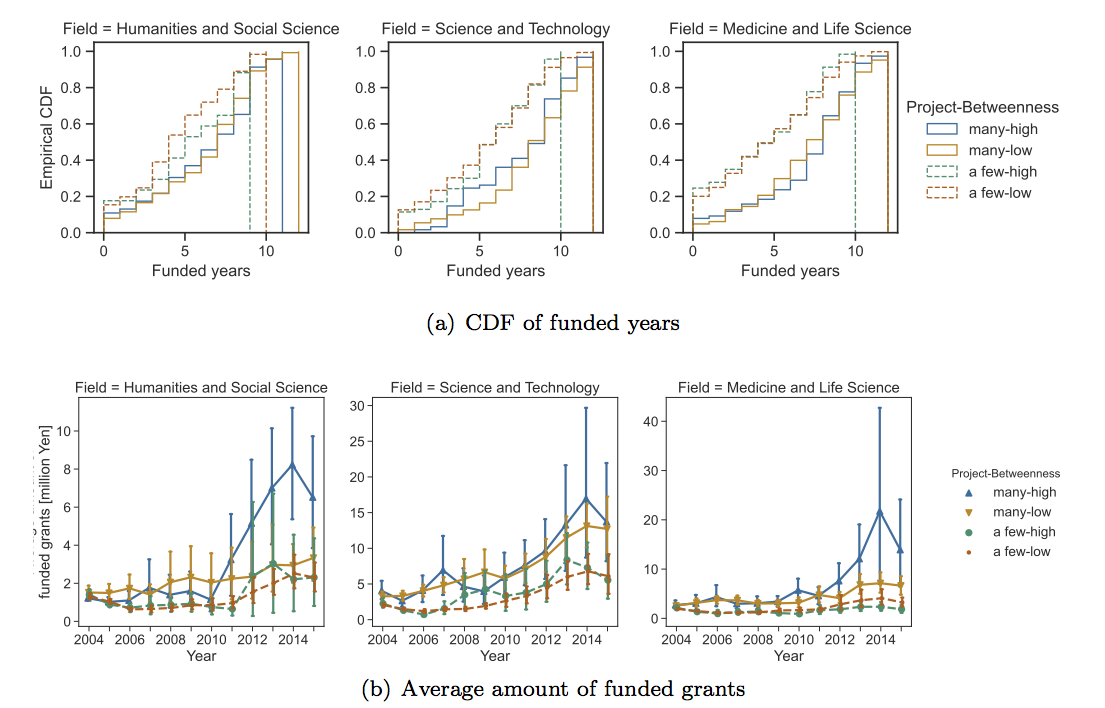

Supplement: S6 Fig — (TIF) [file pone.0277621.s006.tif]

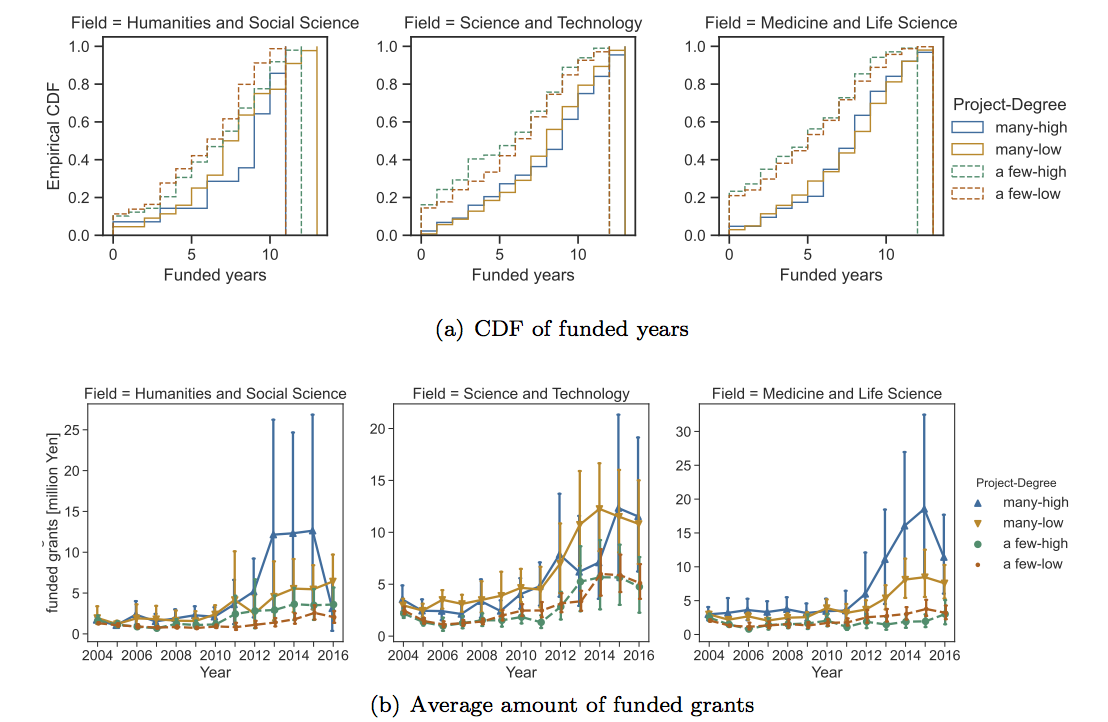

Supplement: S7 Fig — (TIF) [file pone.0277621.s007.tif]

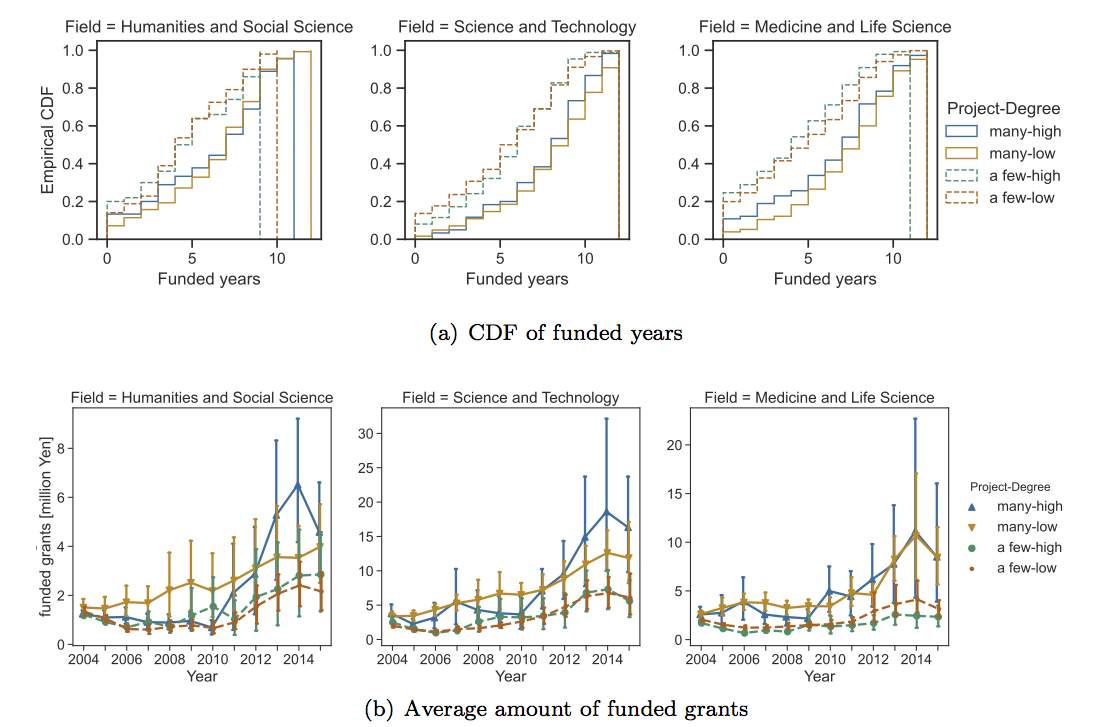

Supplement: S8 Fig — (TIF) [file pone.0277621.s008.tif]
